# *opp*-Dibenzoporphyrin Pyridinium Derivatives as Potential G-Quadruplex DNA Ligands

**DOI:** 10.3390/molecules28176318

**Published:** 2023-08-29

**Authors:** Nuno M. M. Moura, José A. S. Cavaleiro, Maria Graça P. M. S. Neves, Catarina I. V. Ramos

**Affiliations:** LAQV-REQUIMTE, Department of Chemistry, University of Aveiro, 3810-193 Aveiro, Portugal; jcavaleiro@ua.pt (J.A.S.C.); gneves@ua.pt (M.G.P.M.S.N.)

**Keywords:** dibenzoporphyrins, G-quadruplexes, telomerase inhibition, selectivity, UV-Vis, fluorescence, G4-FID, circular dichroism

## Abstract

Since the occurrence of tumours is closely associated with the telomerase function and oncogene expression, the structure of such enzymes and genes are being recognized as targets for new anticancer drugs. The efficacy of several ligands in telomerase inhibition and in the regulation of genes expression, by an effective stabilisation of G-quadruplexes (G4) DNA structures, is being considered as a promising strategy in cancer therapies. When evaluating the potential of a ligand for telomerase inhibition, the selectivity towards quadruplex versus duplex DNA is a fundamental attribute due to the large amount of double-stranded DNA in the cellular nucleus. This study reports the evaluated efficacy of three tetracationic *opp*-dibenzoporphyrins, a free base, and the corresponding zinc(II) and nickel(II) complexes, to stabilise G4 structures, namely the telomeric DNA sequence (AG_3_(T_2_AG_3_)_3_). In order to evaluate the selectivity of these ligands towards G4 structures, their interaction towards DNA calf thymus, as a double-strand DNA sequence, were also studied. The data obtained by using different spectroscopic techniques, such as ultraviolet-visible, fluorescence, and circular dichroism, suggested good affinity of the free-base porphyrin and of its zinc(II) complex for the considered DNA structures, both showing a pattern of selectivity for the telomeric G4 structure. A pattern of aggregation in aqueous solution was detected for both Zn(II) and Ni(II) metallo dibenzoporphyrins and the ability of DNA sequences to induce ligand disaggregation was observed.

## 1. Introduction

Chromosomic and genomic stability is ensured by the presence of special structures at each of the chromosome ends, the telomeres. The presence of these structures protects the chromosome ends from enzymatic degradation [1,2,3,4], and their main functions are as follows: (i) to guarantee that the genetic information is perfectly copied during cell duplication, (ii) to maintain the stability of chromosome structure, and (iii) to prevent the fusion of consecutive chromosome ends and genetic mutations that can culminate with the appearance of tumours or with the degradation of deoxyribonucleic acid (DNA) [5].

Telomeres shortening occurs during each DNA replication and, in normal somatic cells, when a critical length is reached, telomeres can no longer guarantee chromosome integrity and cell senescence and death occur. A different situation happens in cancer cells due to the presence of telomerase, a reverse transcriptase enzyme, that is able to switch off this shortening process [2,6]. Since this enzyme is highly expressed in a series of cancer cells allowing continuous cell division without telomere shortening, it can be viewed as an attractive therapeutic target [2,4].

Each telomeric DNA terminal presents the sequence repeat TTAGGG, (T_2_AG_3_), and can form non-canonic DNA structures, such as the G-quadruplexes (G4). The high percentage of guanines in this sequence enables their self-assembly in a planar quadrangular arrangement via Hoogsteen hydrogen bonding, with the formation of G-quartets. The establishment of π−π interactions between G-quartets on top of each other will give rise to the formation of G4 structures [7,8]. G4 structures could be also formed in genomic regions like transposable elements (TEs), recombination hotspots, 5′ and 3′ untranslated regions (UTRs), among others [9,10,11,12,13].

The inhibition of telomerase activity by ligands stabilisation of DNA secondary structures, namely of G4 present in telomeric ends, is being pointed out as a promising antitumour strategy [7,8,14,15,16]. Moye et al. [17] reported that telomerase has the ability to recognize and partially unwind G-quadruplexes during the 3′-end extension process. So, the stabilisation of G4 structures by ligand binding will lock the telomere in the G4 arrangement, thus inhibiting telomerase function and stopping telomere lengthening of cancer cells [17,18].

Since telomerase cannot recognize G-quadruplex as substrates [19], the indirect targeting of this enzyme, by the stabilisation of G4 DNA secondary structures with ligands, can be considered a promising strategy [20,21,22,23]; this stabilisation could ensure the telomeres configuration in the quadruplex arrangement inhibiting telomerase function and telomere lengthening. A similar strategy could also be used to inhibit oncogenes expression and, as a consequence, to compromise the transcription process [12,24,25,26,27].

The ability of ligands to stabilise DNA structures, namely G4 structures, resulting in telomerase inhibition and/or in the regulation of gene expression has been evaluated using different biochemical and biophysical methods with promising results [28,29,30,31,32,33,34,35,36]. The G4 recognition by ligands can occur at the ends of the G-tetrad core by outside stacking or end-stacking, by interaction with the backbone (core and loop bases) by groove or loop binding, or by intercalation [37,38]. The most favourable binding modes are the end-stacking and loop and groove binding since such binding modes involve lower energetic costs than an intercalation process [17,23,39]. Interestingly, the intercalation of a ligand in G4 structures has been recently demonstrated for the first time by Plavec et al. [40].

The presence in DNA ligands of heteroaromatic moieties with cationic terminal side chains is a structural feature highlighted as a crucial property for effective ligand binding to the G4 structure. In fact, several ligands, namely aromatic macrocycles or ligands at least containing several aromatic rings (fused or not), have been reported as being able to non-covalently bind not only to DNA but also to RNA quadruplex structures [32,41].

Considering that certain ligands of the tetrapyrrolic macrocycles group, such as porphyrins and analogues, can form adducts with G4, a significant number of studies have been published showing the ability of such types of ligands to inhibit the telomerase activity [42,43,44,45,46,47]. Among them, the 5,10,15,20-tetrakis(1-methylpyridinium-4-yl)porphyrin (**TMePyP**) is one of the most studied porphyrins; it has been described as a potential telomerase inhibitor since it presents a high affinity to G4 structures, although lacking selectivity for G4 over double-stranded (ds) DNA structures [18,34,46,48,49,50]. 

Higher selectivity of ligands for G4 when compared with ds DNA is an important feature since the ligand must be able to distinguish G4 structures in the presence of high amounts of ds DNA in the cell nucleus [51]. Moreover, the presence of positive charges in ligand structures can have a positive contribution to water solubility and to electrostatic interactions with the negative charges of DNA structures. Some of these ligand structural features are usually envisaged as important attributes for telomerase inhibition [47,52,53,54]. 

**Figure 1:**
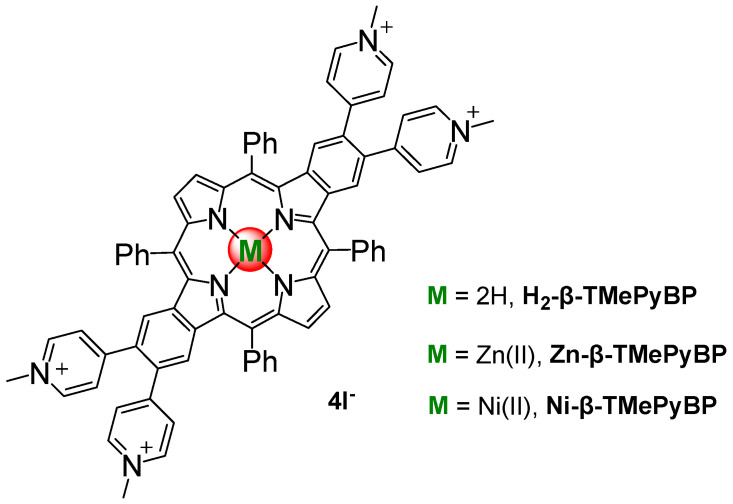
Structures of the studied **β-TMePyBP** derivatives.

Following our interests on the synthesis and potential biological applications, particularly as potential telomerase inhibitors of tetrapyrrolic macrocycles [55,56,57,58,59,60,61], and also considering the ability of cationic porphyrins to stabilise G4 structures, in this study the ability of three related tetracationic *opp*-dibenzoporphyrins—the free base **H_2_-β-TMePyBP** and the corresponding zinc(II) and nickel(II) complexes, **Zn-β-TMePyBP**, and **Ni-β-TMePyBP** (Figure 1) as G4 stabilising ligands was evaluated. The main target of this work is to access how the structural features of these cationic *opp*-dibenzoporphyrins, namely the presence of positive charges in the β-pyrrolic substituents and the higher rigidity of the ligand, are able to influence the capability of such derivatives to stabilise G4 structures.

The efficiency of the selected cationic ligands to stabilise G4 structures, was evaluated by analysing the hypochromic and bathochromic effects caused by the DNA structures in the ligand UV-Vis spectra. The results obtained in these titrations were confirmed using complementary techniques like fluorescence and circular dichroism (CD).

## 2. Results

### 2.1. Synthesis

**Scheme 1:**
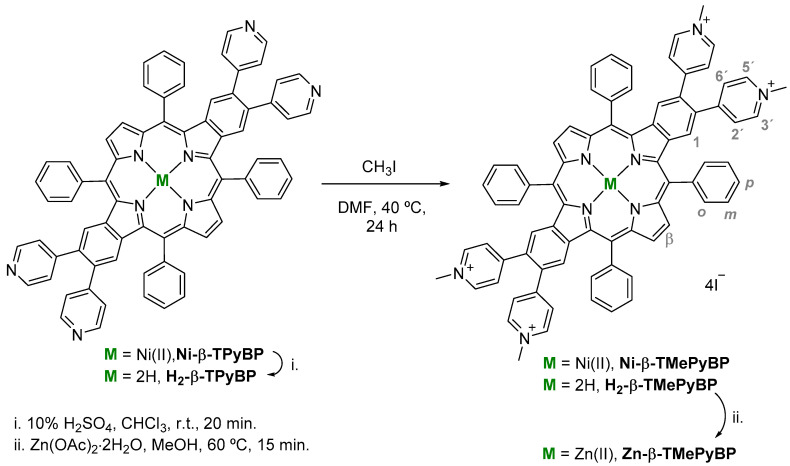
Synthetic approach to prepare tetracationic *opp*-dibenzoporphyrin derivatives **Ni-β-TMePyBP**, **H_2_-β-TMePyBP**, and **Zn-β-TMePyBP**.

The tetracationic *opp*-dibenzoporphyrin derivatives **H_2_-β-TMePyBP**, **Zn-β-TMePyBP**, and **Ni-β-TMePyBP** were prepared as outlined in Scheme 1. The synthetic route used involved the previous preparation of the neutral Ni(II) complex of *opp*-dibenzoporphyrin **Ni-β-TPyBP** through the Heck reaction approach [50,62]. Briefly, the **Ni-β-TPyBP** precursor was prepared from 5,10,15,20-tetraphenylporphyin (**TPP**) throughout a three-step approach that implied the tetra-bromination at two opposite β-pyrrolic positions by using *N*-bromosuccinimide as the brominating agent [63], followed by metalation with Ni(II) [64] and, finally, the palladium-mediated cross-coupling Heck reaction with 4-vinylpyridine under microwave irradiation (Scheme S1) [50,62]. This synthetic strategy gave rise to **Ni-β-TPyBP** scaffold in 72% yield, with a reaction time reduction of more than 85% and a yield improvement of 24% over the conventional heating source. This complex, after treatment with 10% conc. H_2_SO_4_ in CHCl_3_, resulted in the corresponding free-base **H_2_-β-TPyBP** in 98%.

Then, the quaternization of pyridyl units present in **Ni-β-TPyBP** and **H_2_-β-TPyBP** with methyl iodide afforded, after 24 h at 40 °C in DMF, the expected tetracationic *opp*-dibenzoporphyrins **Ni-β-TMePyBP** and **H_2_-β-TMePyBP** in 94% and 97% yield, respectively. The complex **Zn-β-TMePyBP** was obtained in 98% yield by metalation of the free-base **H_2_-β-TMePyBP** with Zn(OAc)_2_·2H_2_O in MeOH.

The structure of each tetracationic *opp*-dibenzoporphyrin derivative **H_2_-β-TMePyBP**, **Zn-β-TMePyBP**, and **Ni-β-TMePyBP** was confirmed by ^1^H and ^13^C nuclear magnetic resonance (NMR), UV-Vis absorption spectroscopy, and mass spectrometry. 

In general, the NMR spectra of the three tetra-cationic *opp*-dibenzoporphyrins present an analogous profile. For each compound, in the aromatic region, the singlet appearing between δ 7.34 ppm to δ 7.03 ppm is ascribed to the resonance of the four protons from the two aromatic rings fused at the porphyrinic core. The pyridinium units generate two duplets (*J* ≈ 6.5 Hz) at approximately δ 8.90 ppm and δ 7.68 ppm due to the resonances of protons H-3″,5″ and H-2″,6″, respectively.

The protons of the phenyl rings at the *meso* positions of the dibenzoporphyrinic ring generate resonances in the range between δ 8.02 ppm and δ 7.86 ppm assigned to the resonances of the *meta* and *para* protons. For **H_2_-β-TMePyBP** and **Zn-β-TMePyBP**, the *ortho* protons from the phenyl rings generate a duplet (*J* ≈ 7.0 Hz) at δ 8.25 ppm and δ 8.20 ppm, respectively. However, for **Ni-β-TMePyBP** the resonances due to the *ortho* protons appear as a multiplet ranging from δ 8.33 ppm to δ 8.03 ppm along with the signal due to the resonance of the four β-pyrrolic protons. For the other two benzoporphyrins, the signals due to β-pyrrolic protons appear as singlets at δ 9.01 ppm for **H_2_-β-TMePyBP** and at δ 8.84 ppm for the complex **Zn-β-TMePyBP**.

The accomplishment of the *N*-methylation with methyl iodide was confirmed by ^1^H NMR with the presence of a singlet at approximately δ 4.35 ppm due to the resonance of the protons from the *N*-methyl groups. Also, the peak at δ 48.1 ppm in their ^13^C NMR spectra due to the *N*-methyl carbon corroborates the success of the alkylation. The ^1^H NMR spectrum of the **H_2_-β-TMePyBP** displays a characteristic singlet at δ −2.68 ppm ascribed to the resonance of the two *N*-H protons in the porphyrinic core.

The three tetracationic *opp*-dibenzoporphyrins show, in their ESI-MS spectra, the expected *m/z* values corresponding to the [M]^4+^ molecular ions.

**Figure 2:**
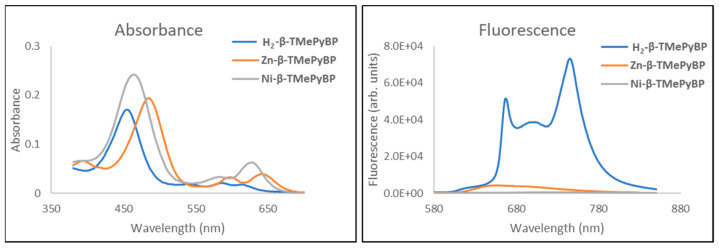
Absorption (**left**) and emission (**right**) spectra of tetracationic *opp*-dibenzoporphyrin derivatives **H_2_-β-TMePyBP**, **Zn-β-TMePyBP** and **Ni-β-TMePyBP** recorded in PBS/DMSO (1%) mixture at 298 K and at 2.0 µM concentration; λ_exc(_**_H2-β-TMePyBP)_** = 454 nm; λ_exc(_**_Ni-β-TMePyBP)_** = 466 nm, λ_exc(_**_Zn-β-TMePyBP)_** = 485 nm).

The absorption and emission spectra of the tetracationic derivatives **H_2_-β-TMePyBP**, **Zn-β-TMePyBP**, and **Ni-β-TMePyBP** were recorded in phosphate buffer saline solution (PBS)/DMSO (1%) medium at a concentration of 2.0 μM and 298 K and are shown in Figure 2.

The **H_2_-β-TMePyBP** derivative displays an absorption spectrum with a strong Soret band centered at 454 nm due to S_0_→S_2_ transitions and three Q bands ranging from 541 to 613 nm ascribed to S_0_→S_1_ transitions [65]. Regarding the absorption spectra of the tetracationic metallo dibenzoporphyrins **Zn-β-TMePyBP** and **Ni-β-TMePyBP**, the Soret band was red-shifted 29 and 12 nm, respectively, when compared with the free-base derivative **H_2_-β-TMePyBP**. Since the metal insertion in the dibenzoporphyrin core leads to an improvement in the symmetry of the molecule [66,67], the UV-Vis spectra of both **Zn-β-TMePyBP** and **Ni-β-TMePyBP** exhibit only two Q bands ranging from 574 to 635 nm.

Unlike what is usually observed for free-base porphyrins derivatives and their metallo complexes that show a sharp Soret band at approximately 420 nm [65,66], the Soret band of tetracationic *opp*-dibenzoporphyrin derivatives **H_2_-β-TMePyBP**, **Zn-β-TMePyBP**, and **Ni-β-TMePyBP** are broader and significantly red-shifted due to their extended π-electronic delocalization presumably due to the two-fused aromatic rings. 

The emission spectrum of **H_2_-β-TMePyBP** present two well-defined emission bands centered at 668 and 747 nm and a broad band with an emission maximum at 702 nm. When the emission spectrum was recorded for **Zn-β-TMePyBP** and **Ni-β-TMePyBP**, a meaningful decrease in the emission intensity was observed for the **Zn-β-TMePyBP** derivative when compared with the free-base **H_2_-β-TMePyBP**, accompanied by a drastic change into the emission profile. The **Zn-β-TMePyBP** derivative displays an emission band with a maximum at 658 nm and a shoulder band at ca. 697 nm. The fluorescence emission spectra recorded for the corresponding **Ni-β-TMePyBP** complex do not exhibit noticeable emission bands after excitation at 466 nm.

### 2.2. DNA Stabilisation Studies

It is well known that the particular spectroscopic spectra of porphyrins allow the study of their interactions with different DNA structures using UV-Vis and other spectroscopic techniques. Thus, the ability of the considered dibenzoporphyrins to stabilise G4 structures, namely the telomeric DNA sequence (AG_3_(T_2_AG_3_)_3_) able to form a G-quadruplex in the unimolecular topology [68], was assessed using such established techniques, namely the UV-Vis and fluorescence spectroscopies, G-quadruplex fluorescent intercalator displacement (G4-FID) assay, and circular dichroism (CD) melting experiments. Calf thymus, a double-strand DNA sequence, with a length of approximately 1kbp was also used in these studies to compare the affinity and to evaluate the selectivity of the tested porphyrins for G4 structures. The sequences and topologies of the studied oligonucleotides are presented in Table 1.

#### 2.2.1. UV-Vis Spectroscopy

Considering the special characteristics of the electronic absorption spectra of porphyrins, it is possible to monitor their interactions with DNA structures using UV-Vis spectroscopy. In general, the most significant alterations occur in their Soret bands and intercalative binding processes are justified by hypochromism values higher than 35% and bathochromic (∆λ) shifts higher than 15 nm; these values were obtained for long double-stranded sequences for which the end stacking is insignificant [44,69]. When outside binding or groove binding takes place, less significant changes in porphyrins UV-Vis absorption spectra (red shifts < 8 nm) are expected since less direct contact between π-systems occurs [70,71].

Thus, the behaviour of the three cationic dibenzoporphyrins in the presence of increasing amounts of the human telomeric sequence AG_3_(T_2_AG_3_)_3_, able to form a G4 structure, and of the double-stranded (ds) calf thymus sequence, was followed by UV-Vis titrations. These titrations were performed by successive additions of each oligonucleotide in PBS to the dibenzoporphyrin solutions in PBS (2.0 μM) and were finished after observing three consecutive values of constant absorbance [69,72]. In the assays performed in the presence of G4 structures, the plateau was reached after the addition of 3 equivalents of DNA. For further comparison, equivalent amounts were added in the titrations with the ds DNA sequence. In the absorbance data obtained for the different titrations, any variation due to the effect of PBS by performing blank experiments using merely PBS was taken into account.

**Figure 3:**
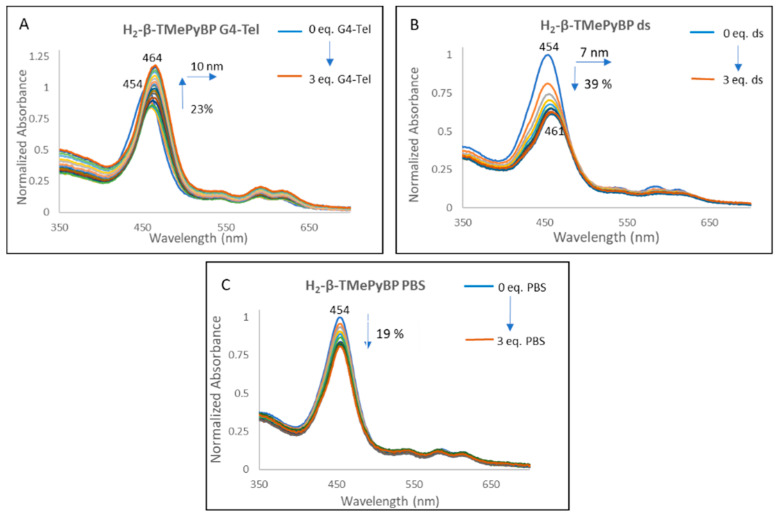
UV-Vis absorption spectra (350–700 nm) for the titrated solutions of **H_2_-β-TMePyBP** (2.0 μM) with (**A**) G4 telomeric, (**B**) double-stranded DNA, and (**C**) PBS buffer.

**Figure 4:**
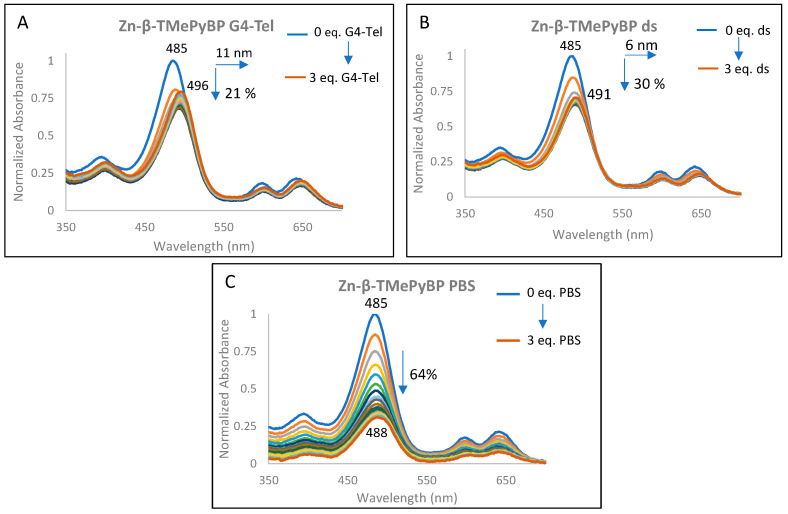
UV-Vis absorption spectra (350–700 nm) for the titrated solutions of **Zn-β-TMePyBP** (2.0 μM) with (**A**) G4 telomeric, (**B**) double-stranded DNA, and (**C**) PBS buffer.

**Figure 5:**
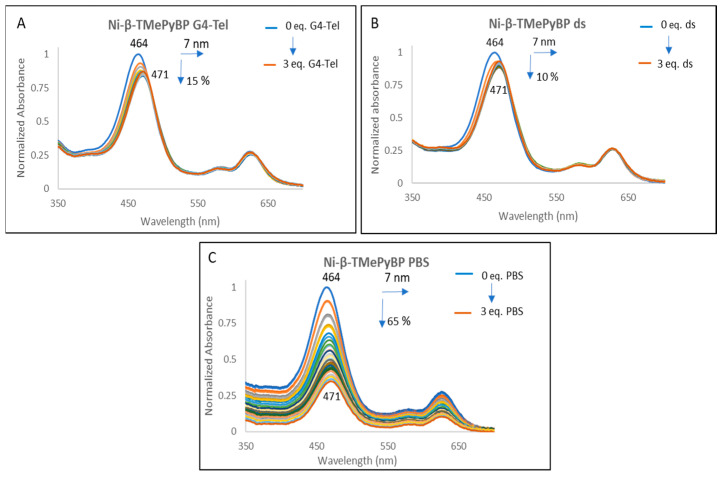
UV-Vis absorption spectra (350–700 nm) for the titrated solutions of **Ni-β-TMePyBP** (2.0 μM) with (**A**) G4 telomeric, (**B**) double-stranded DNA, and (**C**) PBS buffer.

The interactions between the selected DNA sequences and the ligands **H_2_-β-TMePyBP**, **Zn-β-TMePyBP,** and **Ni-β-TMePyBP** were obtained in the range of 350–700 nm and the resulting spectra are presented in Figure 3, Figure 4 and Figure 5, respectively; the titrations just in the presence of PBS are also depicted. For each ligand, the most representative changes occurred in the Soret band region during these titrations, that together with the calculated binding constants, are summarized in Table 2.

The results obtained, at the end of the titration of **H_2_-β-TMePyBP** with the G4 structure (Figure 3A), showed a higher bathochromism (10 nm) along with an hyperchromic effect (23%), when compared to the 7 nm of bathochromism and 39% of hypochromism observed in the case of the calf thymus ds DNA (Figure 3B). In the blank experiment performed with PBS (Figure 3C), only a hypochromic effect of 19% was observed. Considering these results, the higher affinity of the free-base porphyrin for G4 versus calf thymus ds DNA is evident, since at the end of titration the interaction results in a hyperchromic effect of 23%, after 16% of hypochromism observed during the first three DNA addictions. In the case of ds DNA, a lower hypochromic effect of 20% is observed at the end of the titration, pointing to smaller affinity of the ligand for this structure.

The zinc(II) complex **Zn-β-TMePyBP** in the presence of G4 structure (Figure 4A) was responsible for a bathochromism of 11 nm accompanied by a hypochromism of 21%. The interaction of this ligand with the ds structure (Figure 4B), resulted in a smaller red shift deviation of 6 nm and a hypochromism of 30%. In the titration performed with PBS (Figure 4C), a large hypochromism of 64% accompanied by red shift of 3 nm is observed, pointing, probably, to the occurrence of a process of aggregation of the ligand in aqueous medium.

The lower hypochromic effect observed at the end of titrations with DNA structures, when compared with that observed in the PBS titration, could be ascribed to the ability of DNA structures, especially G4, to minimise the occurrence of ligands’ aggregation probably due to their embedment between ligand molecules [69]. The easy involvement of porphyrins and analogs, like phthalocyanines, in stacking or aggregation processes is, in general, justified by their propensity to minimise contact with water due to their hydrophobic core [73]. However, the occurrence of red shifts in the presence of the DNA structures indicates that, along with the disaggregation processes, the interaction of ligands with DNA also occurs. The obtained results point to a pattern of selectivity of this ligand for G4, that results in much-pronounced modifications in the Soret band of this cationic dibenzoporphyrin.

Figure 5 shows the results obtained at the end of each titration with **Ni-β-TMePyBP**. The titration with PBS results in significant changes in the ligand spectra, corresponding to a hypochromic effect of 65% and a red shift of 7 nm, clearly pointing to the occurrence of aggregation processes (Figure 5C).

The results obtained with both DNA structures are similar, showing red shifts of 7 nm and hypochromic effects of 15% for G4 (Figure 5A) and 10% for ds DNA (Figure 5B). Although a lower hypochromism was observed for DNA structures, the same red shift was obtained in titrations with PBS. This behaviour indicates that the DNA structures also have a positive effect in avoiding the occurrence of aggregation of this ligand but its further interaction with DNA seems to be weak. The similarity of the hypochromic effect for both G4 and ds structures also highlights that **Ni-β-TMePyBP** is not selective.

The described results at the end of the different titrations showed, especially for **Zn-β-TMePyBP**, bathochromic deviations considerably higher for G4 when compared with the double-stranded structure (redshift 11 nm vs. 6 nm; hypochromism 21% vs. 30%). These data suggest that the ligand with higher stabilisation ability and strong interaction with G4 is the zinc(II) complex which also shows a pattern of selectivity.

The results from UV-Vis titrations were also used to obtain the binding constants (K_b_) (Table 2) and the binding stoichiometries by applying the method of continuous variations (see experimental section) [18]. The results showed, for all ligands, the occurrence of interactions in a 9:1 stoichiometry (Figure S7). The values of the binding constants ranged from 3.71 × 10^6^ to 7.05 × 10^6^ in the case of G4, and from 1.42 × 10^6^ to 3.46 × 10^6^ for ds DNA. The obtained constants confirm the higher selectivity of the free-base dibenzoporphyrin (K_bG4_ ~ 4 × K_bds_) and of the zinc(II) complex (K_bG4_ ~ 1.9 × K_bds_) towards telomeric G4 vs. ds DNA. In the case of the nickel(II) complex, the similarity of the obtained constants (K_bG4_ ~ K_bds_) confirms the pattern of non-selectivity of this complex.

The unusual high binding stoichiometry obtained can be attributed to the presence of multiple electrostatic ligand binding modes. These modes include end-stacking, loop binding, and groove binding, the latter two being a result of side arm substituents at the beta pyrrolic positions.

Porphyrinic-based ligands possess a broad aromatic surface that facilitates stacking interactions with the external G-quartets of the G-quadruplex. Additionally, *opp*-dibenzoporphyrins have a rigid structure that maximises quartet overlap while obstructing the intercalation process. Furthermore, their cationic charges enable electrostatic interactions to take place with the negatively charged biopolymer [74]. Based on the obtained data and well-known knowledge [18,34], it is reasonable to suggest that the ligand–DNA binding predominantly occurs through the end-stacking of *opp*-benzoporphyrins in the terminal G-quartets, a phenomenon previously observed in studies employing tetracationic *meso*-substituted porphyrins.

On the other hand, it is important to consider that the interaction resulting from the end-stacking of nine positively charged molecules could lead to significant repulsion due to the size of the molecules and the repulsive effect between the four positive charges of each molecule. Therefore, it is plausible that other electrostatic interactions in G4 loops and grooves, which could be potentially favoured by the presence of cationic pyridyl substituents distanced from the porphyrinic nucleus, might occur. *opp*-Dibenzoporphyrin self-association must be also considered, given the stoichiometry obtained. 

#### 2.2.2. Fluorescence Experiments

The intrinsic fluorescence of ligands is an interesting advantage when performing fluorimetric titrations and is one of the ligand properties selected for the present study. Thus, to confirm the results obtained from the UV-Vis experiments, the free-base, zinc(II), and nickel(II) complexes of the dibenzoporphyrins were also studied using fluorescence experiments.

**Figure 6:**
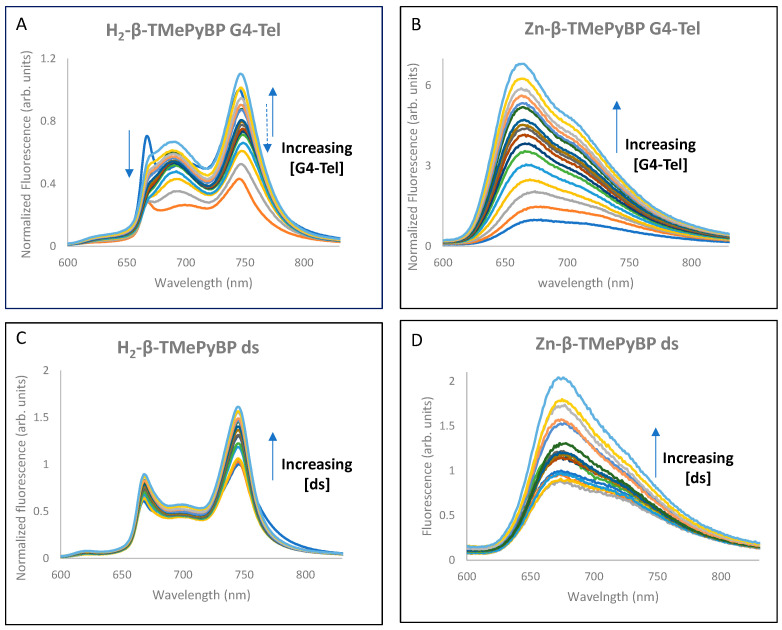
Fluorescence emission spectra of (**A**) **H_2_-β-TMePyBP** with increasing concentration of telomeric G4, (**B**) **Zn-β-TMePyBP** with increasing of telomeric G4, (**C**) **H_2_-β-TMePyBP** with increasing concentration of double-strand (ds), and (**D**) **Zn-β-TMePyBP** with increasing concentration of double-strand (ds).

Considering that **Ni-β-TMePyBP** is not emissive (see Figure 2), the fluorescence studies were only performed in the presence of the emissive **H_2_-β-TMePyBP** and **Zn-β-TMePyBP** ligands. The spectra obtained at the end of the titrations of each ligand with both DNA structures are presented in Figure 6 and were normalized to facilitate the analysis and discussion.

Interestingly, both the free-base and the zinc(II) complex showed a “turn-on” fluorescence during titration with both the telomeric G4 and calf thymus ds DNA. Similar effects have already been observed for cationic phthalocyanines upon interaction with c-MYC G4 [75] and telomeric G4 [73]. Figure 6A,B shows that, at the end of titration with telomeric G4, the maximum enhancement of the fluorescence intensity in the case of the zinc(II) complex (from 1.04 to 6.80) is almost six times higher than the one observed for **H_2_-β-TMePyBP** (from 1.0 to 1.18, after a decrease of ~0.60 with the first G4 addition). These results are in accordance with the disaggregation of ligands in the presence of DNA structures and also point to a higher affinity of **Zn-β-TMePyBP** for the G4. When comparing the intensity obtained in the titrations with the ds DNA (Figure 6C,D), the increase in fluorescence was similar for both ligands (from 1 to 1.6 in the case of the free base and 1.0 to 2.2 for zinc complex). The spectrofluorimetric titrations of the zinc(II) complex with G4 and ds structures confirmed the pattern of selectivity attained in the UV-Vis titrations with the same DNA structures previously discussed.

The G-Quadruplex fluorescent intercalator displacement (G4-FID) assay is another well-established fluorescence-based methodology, extensively used to evaluate the affinity of ligands to G4 and to confirm their selectivity pattern for G4 structures. The G4-FID assay takes advantage of the loss of fluorescence of the thiazole orange (TO) probe resulting from its displacement from DNA by a ligand, when the ligand shows higher affinity for the studied G4 [76,77]. The ligand concentration required to displace 50% of TO from the adduct TO-G4 or TO-ds, giving rise to a decrease in the fluorescence of the probe (TO) by 50%, is noted by DC_50_.

**Figure 7:**
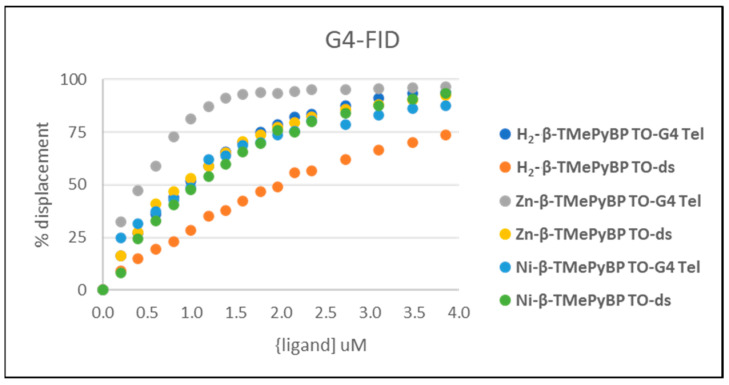
G4-FID assay performed with **H_2_-β-TMePyBP**, **Zn-β-TMePyBP**, and **Ni-β-TMePyBP** and the TO adducts pre-formed with telomeric G4 (TO-G4) and double-stranded DNA (TO-ds).

To further confirm and validate the previous UV-Vis and fluorescence titrations obtained data, the ability of **H_2_-β-TMePyBP**, **Zn-β-TMePyBP**, and also of **Ni-β-TMePyBP** to displace TO from TO-G4 and also from TO-ds adducts, was evaluated by G-Quadruplex fluorescent intercalator displacement (Figure 7 and Figures S8–S10, Table 3). 

**Zn-β-TMePyBP** (0.42 μM) presents the lowest DC_50_ value for the TO-G4 adducts followed by the free-base (0.99 μM) and then the Ni(II) complex (1.04 μM). For the first two ligands, the values obtained are almost 50% lower than the ones observed for the TO-ds adducts (0.93 μM for **Zn-β-TMePyBP** and 1.99 for the free base). These G4-FID data agree with the results obtained from UV-Vis and fluorescence titrations and confirm the pattern of good affinity and selectivity of both ligands for the G4 DNA. Similar to the previous assays, these results point out again the higher affinity of **Zn-β-TMePyBP** for G4 when compared to the free-base dibenzoporphyrin **H_2_-β-TMePyBP**.

The DC_50_ values obtained with the nickel complex in the presence of TO-G4 (1.04 μM) and TO-ds (1.02 μM) corroborate the pattern of less affinity and non-selectivity of this ligand towards both DNA structures previously found.

#### 2.2.3. Circular Dichroism (CD)

Circular dichroism (CD) is another spectroscopic technique that provides important information regarding the efficiency of a ligand to stabilise G4 DNA structures, namely by evaluating the melting temperature (T_m_) of a G4 sequence in the presence and in the absence of a ligand [78]. 

Prior to the melting assay, the CD Spectra of the oligonucleotide G4-Tel and of the adducts obtained with the different ligands were obtained in order to verify if the quadruplex structure is maintained in the presence of ligands. The obtained spectra are shown in Figure S11. As can be seen, the spectra of the G4-tel present two characteristic bands, a positive one at ≈ 295 nm and a negative band at ≈ 240 nm, which is typical of hybrid G4.

In the presence of the ligands, the G4 maintains its conformation and no significative alterations are observed, indicating the stabilisation effect of the ligands. 

**Figure 8:**
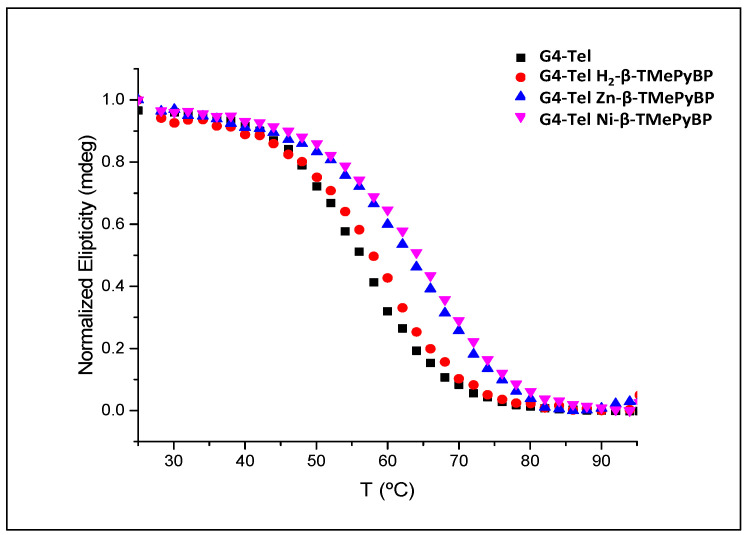
CD melting profiles obtained for telomeric G4 (G4-Tel) in the absence and the presence of 2 equivalents of **H_2_-β-TMePyBP**, **Zn-β-TMePyBP**, and **Ni-β-TMePyBP**.

The difference between the obtained melting values, ΔT_m_, in the presence and the absence of a ligand reveals the stabilisation of the G4 structure induced by the presence of a ligand. The melting profiles obtained for the human telomeric G4 in the absence and in the presence of the three ligands **H_2_-β-TMePyBP**, **Zn-β-TMePyBP**, and **Ni-β-TMePyBP** are presented in Figure 8 and the T_m_ and ΔT_m_ values obtained are summarized in Table 4. The results show that the cationic dibenzoporphyrins are able to stabilise the G4 telomeric structure being the highest stabilisation (higher ΔT_m_ values) observed with both complexes. 

The lower melting temperature observed for the adduct G4-**H_2_-β-TMePyBP** (T_m_ = 58.4 °C) when compared with that obtained for G4-**Zn-β-TMePyBP**, (T_m_ = 63.5 °C) is in agreement with the results obtained and described with the other spectroscopic experiments and points to a high stabilisation ability of the zinc(II) complex. The unexpected result obtained for the nickel(II) complex results probably from the temperature gradient, 25 to 95 °C, that occurs during the CD melting temperature technique. An increase in the temperature certainly promotes the disaggregation of the ligand, which will thus become available to interact with the G4 structure. This result seems to point out that, in a situation of non-aggregation, the nickel(II) complex also has the ability to stabilise G4.

## 3. Materials and Methods

### 3.1. General Remarks

5,10,15,20-Tetraphenylporphyrin (**TPP**) [79,80], 2,3,12,13-tetrabromo-5,10,15,20-tetraphenylporphyrin (**β-Br_4_TPP**), and its Ni(II) complex (**Ni-β-Br_4_TPP**) were prepared by following previously described methods [63,64]. Solvents were purified or dried by common laboratory methods [81]. All reagents were obtained from Aldrich (Lisbon, Portugal), Fluka (Gillingham, UK), Alfa Aesar (Heysham, Lancashire, UK), or Acros (Verona, Italy) and used as supplied. MW-assisted synthesis was carried out using a monomode microwave CEM Discover SP instrument (CEM Corporation, Matthews, NC, USA). Column chromatography was carried out on silica gel (Merck, 35–70 mesh, Darmstadt, Germany). TLC was carried out on aluminium-backed Kieselgel plates (Merck 60, 0.2 mm thick). The NMR spectra were recorded either with Bruker Avance 300 (^1^H: 300.13 MHz, Bruker, Wissembourg, France) or with Bruker Avance 500 (^1^H: 500 MHz and ^13^C: 125 MHz) instruments in CDCl_3_ or DMSO-*d*_6_ as solvents; the chemical shifts are expressed in δ (ppm) and the coupling constants (*J*) in Hertz (Hz). Mass spectra were recorded with a Micromass Q-Tof 2 instrument (Micromass, Manchester, UK), operating in the positive ion mode. The UV-Vis spectra were recorded on an UV-2501 PC Shimatzu spectrophotometer (Shimatzu, Kyoto, Japan) and fluorescence emission spectra were recorded on a Horiba Jobin-Yvon Fluoromax 4 spectrofluorimeter (Shimatzu, Kyoto, Japan), using PBS/DMSO (1%) as the solvent.

### 3.2. Synthesis of Macrocyclic Ligands

#### 3.2.1. Synthesis of Ni(II) Complex **Ni-β-TPyBP** and Its Corresponding Free-Base **H_2_-β-TPyBP**

**Ni-β-Br_4_TPP** was prepared by following a synthetic approach previously described [64]; however, in the present case, looking for a potential reaction improvement (reaction time and yields), instead of using the classical heating conditions, a microwave irradiation was used as the heating source (250 W at 120 °C for 5 min). The free-base **H_2_-β-TPyBP** derivative was prepared by acidic treatment of **Ni-β-TPyBP** following a well-established protocol [82]. The structure of both derivatives was confirmed by ^1^H NMR spectroscopy and mass spectrometry and the results agreed with data reported in the literature [50].

#### 3.2.2. Synthesis of Free-Base Derivative **H_2_-β-TPyBP**

To a solution of **Ni-β-TPyBP** (35 mg, 33.2 µmol) in chloroform (5 mL) under stirring and at room temperature, 0.5 mL of conc. sulfuric acid was added. After 20 min, a saturated aqueous solution of NaHCO_3_ was added to neutralize the reactional mixture. Then, the mixture was washed with distilled water, extracted with dichloromethane, and the organic phase was evaporated to dryness under reduced pressure. Pure **H_2_-β-TPyBP** was obtained in 98% and its structure was confirmed by ^1^H NMR spectroscopy and mass spectrometry.

**^1^H NMR** (300 MHz, CDCl_3_): δ 8.89–8.81 (2H, m, H-β), 8.76–8.70 (2H, m, H-β), 8.57–8.41 8H, m, H-*o*-Py), 8.28–8.11 (8H, m, H-*o-*Ph), 7.95–7.64 (12H, m, H-*m*,*p-*Ph), 7.16–6.87 (12H, m, H-1 and H-*m*-Py), and -2.62 (2H, s, *N*-H) ppm. **MS-ESI(+)**: *m*/*z* 1023.6 [M + H]^+^.

#### 3.2.3. *N*-Methylation of **Ni-β-TMePyBP** and **H_2_-β-TMePyBP**

Into a sealed tube was added **Ni-β-TPyBP** (25 mg, 23.2 µmol) or **H_2_**-**β-TPyBP** (25 mg, 24.4 µmol), methyl iodide (60 equiv.), and DMF (1.0 mL). The reaction was placed under magnetic stirring at 40 °C for 23 h. After cooling to room temperature, diethyl ether was added to the reaction. The precipitate obtained was filtered, washed with diethyl ether, and dissolved with a CH_2_Cl_2_/MeOH (95:5) mixture. The solvent was evaporated to dryness under reduced pressure. **Ni-β-TMePyBP** and **H_2_-β-TMePyBP** were obtained pure in 94% and 97% yield, respectively, after recrystallization in a CH_2_Cl_2_/Hexane (95:5) mixture.

**Ni-** **β** **-TMePyBP**

**^1^H NMR** (500 MHz, DMSO-*d*_6_): 8.86 (8H, d, *J* = 6.5 Hz, H-3″,5″), 8.33–8.03 (12H, m, H-β and H-*o*-Ph), 7.99–7.86 (12H, m, H-*m*,*p*-Ph), 7.69 (8H, d, *J* = 6.5 Hz, H-2″,6″), 7.34 (4H, s, H-1), 4.34 (12H, s, *N-*CH_3_) ppm. **^13^C NMR** (125 MHz, DMSO-*d*_6_): δ 156.1, 145.8, 137.2, 136.4, 136.19, 133.6, 129.9, 129.3, 128.4, 128.2, 123.2, 48.1 ppm. **UV-Vis** (PBS): λ_máx_ (ε) nm = 466 (113,790), 574 (15,880), 622 (29,947) nm. **MS-ESI(+)**: *m/z* 284.7 [M]^4+^.

**H_2_-** **β** **-TMePyBP**

**^1^H NMR** (500 MHz, DMSO-*d*_6_): δ 9.01 (4H, s, H-β), 8.91 (8H, d, *J* = 6.6 Hz, H-3″,5″), 8.25 (8H, d, *J* = 6.9 Hz, H-*o*-Ph), 8.02–7.94 (12H, m, H-*m*,*p*-Ph), 7.68 (8H, d, *J* = 6.6 Hz, H-2″,6″), 7.03 (4H, s, H-1), 4.37 (12H, s, *N-*CH_3_), −2.68 (2H, s, *N*-H) ppm. **^13^C NMR** (125 MHz, DMSO-*d*_6_): δ 155.2, 148.4, 145.8, 142.5, 141.3, 138.9, 133.8, 133.0, 129.9, 129.2, 128.9, 128.5, 128.0, 119.6, 48.1 ppm. **UV-Vis** (PBS): λ_máx_ (ε) nm = 454 (85,350.0), 541 (7937.4), 588 (8651.4), 613 (7595.5) nm. **MS-ESI(+)**: *m/z* 270.7 [M]^4+^.

#### 3.2.4. Synthesis of Zn(II) Complex **Zn-β-TMePyBP**

To a round bottom flask **H_2_-β-TMePyBP** (10 mg, 6.29 μmol), 1.5 equivalents of zinc acetate (2.1 mg, 9.43 μmol) and methanol (2 mL) were added. The mixture was stirred at 60 °C until the total consumption of the starting porphyrin was verified by TLC control. The zinc(II) complex was precipitated with the addition of diethyl ether and centrifuged at 12,000 rotations for 3 min to separate the precipitate from the supernatant. The desired Zn(II) complex **Zn-β-TMePyBP** was attained in 98% yield after recrystallization in a CH_2_Cl_2_/Hexane (95:5) mixture.

**^1^H NMR** (500 MHz, DMSO-*d*_6_): δ 8.88 (8H, d, *J* = 6.4 Hz, H-3″,5″), 8.84 (4H, s, H-β), 8.20 (8H, d, *J* = 7.1 Hz, H-*o*-Ph), 8.01–7.87 (12H, m, H-*m*,*p*-Ph), 7.68 (8H, d, *J* = 6.4 Hz, H-2″,6″), 7.17 (4H, s, H-1), 4.36 (12H, s, *N*-CH_3_), ppm. **^13^C NMR** (125 MHz, DMSO-*d*_6_): δ 155.4, 150.4, 145.7, 143.5, 142.9, 140.6, 133.4, 132.3, 132.0, 129.4, 128.6, 128.5, 128.4, 120.1, 48.1 ppm. **UV/Vis** (PBS): λ_máx_(ε) nm = 485 (97,404), 595 (16,671), 635 (18,548) nm. **MS-ESI(+)**: *m/z* 286.2 [M]^4+^.

### 3.3. Preparation of DNA Structures (Double Chain and G-Quadruplexes)

A PBS solution, containing 20 mM of phosphate buffer (10 mL of KH_2_PO_4_ 1 M, and 200 μL of K_2_HPO_4_ 1 M) and 100 mM of KCl was prepared with pH adjusted to 6.8. The resultant PBS solution was used as the solvent for oligonucleotide solutions. After solubilisation in PBS, each oligonucleotide was heated up to 85 °C for 10 min and left to cool overnight to assure the correct folding into G4 structures. The solutions were stored at −20 °C. In the case of calf thymus, the lyophilized was dissolved in water (1 mg/mL) with no sonication or stirring and keeping a gentle inversion overnight at −4 °C to completely solubilize the DNA. Calf thymus solution have been then stored at 4 °C.

For calf thymus, the sample concentrations were determined by measuring the absorbance of a diluted solution at 260 nm (A_260_), to avoid inaccuracies A_260_ ≤ 1.2. The concentration was then calculated using the formula: Concentration (µg/mL) = A_260_ × dilution factor × weight per OD of stock solution (in µg/OD). The obtained values were then converted to micromolar data for further analysis.

In the case of the G-quadruplex solutions, stock solutions of 1 mM were prepared using MiliQ water. Diluted solutions were then prepared in PBS.

### 3.4. Spectroscopic Methods

#### 3.4.1. UV-Vis Spectroscopy

UV-Vis absorption spectra were acquired in a Shimadzu UV-2501-PC spectrophotometer, using a reduced quartz cuvette 1 cm in length. The ligands were dissolved in PBS to mimic the physiological conditions. Titrations were performed by the addition of cumulative volumes of the DNA solutions (G4 or ds) to 1 mL solution of the dibenzoporphyrins with the initial concentration of 2 μM, in the range of 350–700 nm. Titrations were finished after observing three consecutive values of constant absorbance [69,72]. Blank assays were carried out by adding small aliquots of PBS buffer to each *opp*-dibenzoporphyrin ligand until reaching the cumulative volume corresponding to the addition of 3 equivalents of each DNA structure studied.

To ensure the reproducibility of results, all the experiments were performed in triplicate. The percentage of hyper/hypochromicity of the absorption band was determined using the equation % hypochromicity = [(ε_free_ − ε_bound_)/ε_free_] × 100, where ε_bound_ was calculated using Beer’s Law (ε_bound_ = A_bound_/C_bound_) and ε_free_ is the molar extinction coefficient values experimentally obtained, in PBS.

The binding constants (Kb) were determined by fitting data obtained from the UV-Vis absorption titrations to the equation:

(1) $$\frac{\left[DNA\right]}{\varepsilon_{a-}\varepsilon_{b}}=\frac{\left[DNA\right]}{\varepsilon_{a-}\varepsilon_{b}}+\frac{1}{K_{b}(\varepsilon_{a-}\varepsilon_{b})}$$

where [DNA] is the concentration of DNA, and ε_a_ and ε_b_ correspond to apparent extinction coefficients for the complex in the absence and also fully bound DNA, respectively. The K_b_ was then calculated from the ratio of the slope to the intercept.

The binding stoichiometries were obtained by using the method of continuous variation, or Job plot [83,84]. Job diagrams were obtained by plotting F(χ) against the molar fraction of the ligand (χ) at each point of the titration, according to the following equation:

(2) $$F(\chi )=\frac{A_{obs}-(C_{lig}.\varepsilon_{lig}+C_{DNA}.\varepsilon_{DNA})}{C_{lig}+C_{DNA}}$$

where A_obs_ corresponds to the absorbance observed after each DNA addition at the ligand maximum wavelength, C_lig_ and C_DNA_ correspond, respectively, to the total porphyrin and DNA concentrations after each DNA addition, and ε_lig_ and ε_DNA_ are the extinction coefficients. The extinction coefficients of the DNA structures are: ε_G4-Tel_ = 228.500 M^−1^ cm^−1^ and ε_CT_ = 13.200 M^−1^ cm^−1^ (base pair) at 260 nm [85]. The stoichiometry corresponds to the plot minimum observed. The fraction of the ligand (χ) was calculated using the formulae $\chi =\frac{C_{lig}}{\left[C_{lig}+C_{DNA}\right]}$ at each point of the titration.

#### 3.4.2. Fluorescence Studies 

Fluorescence titrations were conducted using a Horiba Floromax-4 spectrofluorometer. The studied ligands were excited at their maximum absorption wavelength; the collection of the fluorescence emission data took place between 600–850 nm at 25 °C using a 1 cm quartz cuvette containing a 2 μM ligand solution. Our setup involved 5 nm excitation and emission slits.

For G4-FID experiments, stock solutions of 35 μM of TO and 10 μM of each oligonucleotide were prepared. Mixing these solutions produced the desired TO-DNA adduct, and after 10 min of orbital shaking, the fluorescence using a Fluoromax-4 spectrofluorometer (Horiba) was measured. The excitation wavelength was set at 485 nm, and the emission range was at 510–750 nm, with 10 nm slits for both excitation and emission.

The initial fluorescence data were termed F_A0_. To investigate further, solutions with increasing concentrations of each ligand (ranging from 0 to 4 μM) were prepared, which were added to the TO-DNA solutions (17.5 μM TO–5 μM DNA). Again, we measured the fluorescence using the same experimental parameters. The percentage of displacement, DC_50_, was calculated using the following equation:

(3) $${DC}_{50}=100-\frac{F_{A}}{F_{A_{0}}}\times 100$$

where, F_A_ is the fluorescence intensity of each sample minus the background fluorescence from mili-Q water (F_A_ = F − F_H2O_), and F_A0_ corresponds to the fluorescence intensity of the fluorescent probe bound to DNA without the added ligand minus the background fluorescence from mili-Q water (F_A0_ = F_0_ − F_H2O_).

#### 3.4.3. Circular Dichroism 

CD experiments were performed in a Jasco J-1500 spectrophotometer, equipped with temperature controller Jasco PTC-517. Prior to analysis, the equipment was purged with pure nitrogen gas. The solutions containing the DNA structures and the ligands (4 μM) were previously prepared by heating the solution for 10 min at 85 °C and then cooling it overnight. The CD melting spectra were then obtained by monitoring the ellipticity at 295 nm in the temperature range of 25–95 °C. The equation below was used to convert the data obtained into fraction folded (θ) plots [78]:

(4) $$ϴ=\frac{CD-{CD}^{min}}{{CD}^{max}-{CD}^{min}}$$

where, CD is the ellipticity of the monitored wavelength at each temperature, CD^max^ is the highest ellipticity, and CD^min^ is the lowest ellipticity.

The melting temperatures were then obtained by fitting sigmoidal curves with the Origin 8.0 software and the Boltzmann function. 

## 4. Conclusions

In this study, ultraviolet-visible, fluorescence, and circular dichroism spectroscopic methods were used to evaluate the ability of the three cationic *opp*-dibenzoporphyrins **H_2_-β-TMePyBP**, **Zn-β-TMePyBP**, and **Ni-β-TMePyBP** to selectively stabilise G4 DNA structures versus double-stranded DNA structures. The data obtained from the different analytical techniques were in general consistent and it suggests that the free-base **H_2_-β-TMePyBP** and the zinc(II) complex **Zn-β-TMePyBP** have a high affinity and good selectivity for the telomeric G-quadruplex over double-stranded DNA structures. In the case of both complexes, a pattern of aggregation in aqueous solution was observed, which apparently can be reversed through a DNA-promoted ligand disaggregation process. 

The data from the UV-Vis supports, for all the ligands, the formation of ligand–DNA adducts and the obtained binding constants confirm the higher affinity (Kb ≥ 10^6^ M^−1^) and selectivity of the free-base cationic dibenzoporphyrin (K_bG4_ ~ 4 × K_bds_) and of its zinc(II) complex (K_bG4_ ~ 1.9 × K_bds_) towards telomeric G4 versus ds DNA. In the case of the nickel(II) complex, the similarity of Kb_G4_ and Kb_ds_ values confirm the pattern of non-selectivity of this complex.

A “turn-on” fluorescence process was observed for the free-base and zinc(II) complexes during the spectrofluorimetric titrations, which is in line with the disaggregation process of ligands in the presence of DNA structures and also pointing to a higher affinity of **Zn-β-TMePyBP** for the G4. The difference obtained at the end of spectrofluorimetric titrations with the G4 and ds structures in the case of **Zn-β-TMePyBP** confirmed the pattern of selectivity already highlighted by the data from the spectrophotometric titrations. Overall, the obtained results from the UV-Vis and fluorescence spectroscopy, are indicative of the ligand binding towards G-quadruplexes, probably through an end-stacking process, similar to the one described for cationic *meso*-substituted porphyrins. However, electrostatic interactions in G4 loops and grooves, and ligand self-association must be also considered, considering the unusual stoichiometry obtained.

The melting profiles obtained for the human telomeric G4 in the absence and in the presence of all the selected ligands also confirmed that cationic dibenzoporphyrins are able to stabilise the G4 telomeric.

## Figures and Tables

**Table 1:** Sequence and topology of studied oligonucleotides.

Oligonucleotide Sequence	Topology	Abbreviation
5′-AGG GTT AGG GTTAGG GTT AGGG-3′ (human telomeric repeat)	Unimolecular G-Quadruplex	AG_3_(T_2_AG_3_)_3_
long DNA strand (Calf Thymus)	Double strand DNA	ds

**Table 2:** Red shifts, hyperchromic/hypochromic percentages, and binding constants obtained at the end of UV-Vis titrations.

Ligand		Telomeric G4	Calf Thymus (ds)	PBS
**H_2_-β-TMePyBP**	Red Shift (nm)	10	7	0
	(%) Hypo/Hyper chromism	+23	−39	−19
	K_b_ (M^−1^)	6.01 ± 1.11 × 10^6^	1.42 ± 0.47 × 10^6^	---
**Zn-β-TMePyBP**	Red Shift (nm)	11	6	3
	(%) Hypo/Hyper chromism	−21	−30	−64
	K_b_ (M^−1^)	7.05 ± 1.31 × 10^6^	3.17 ± 0.31 × 10^6^	---
**Ni-β-TMePyBP**	Red Shift (nm)	7	7	7
	(%) Hypo/Hyper chromism	−15	−10	−65
	K_b_ (M^−1^)	3.71 ± 1.28 × 10^6^	3.46 ± 0.89 × 10^6^	---

Note: The “+” and “−” represent hyperchromism and hypochromism, respectively.

**Table 3:** DC_50_ values obtained for studied ligands in PBS at 25 °C.

	DC_50_ (μM)		$\frac{{DC}_{50}ds}{{DC}_{50}G4}$
	TO-ds	TO-G4	
**H_2_-β-TMePyBP**	1.99 ± 0.26	0.90 ± 0.04	2.01
**Zn-β-TMePyBP**	0.93 ± 0.18	0.42 ± 0.08	2.21
**Ni-β-TMePyBP**	1.04 ± 0.12	1.02 ± 0.06	1

**Table 4:** CD melting temperatures.

	T_m_ (°C)	ΔT_m_ (°C)
G4-Tel	56.1 ± 1.4	---
G4-Tel + **H_2_-β-TMePyBP**	58.4 ± 1.8	2.3
G4-Tel + **Zn-β-TMePyBP**	63.5 ± 1.3	7.4
G4-Tel + **Ni-β-TMePyBP**	64.6 ± 1.6	8.5

## Data Availability

The data that support the findings of this study are available from the corresponding authors upon reasonable request.

## Supplementary Materials

The following supporting information can be downloaded at: https://www.mdpi.com/article/10.3390/molecules28176318/s1, Scheme S1: Synthetic route to prepare the neutral Ni(II) complex of *opp*-dibenzoporphyrin **Ni-β-TPyBP**; Figures S1–S6: NMR spectra; Figure S7: Job plot obtained from UV-Vis titrations; Figures S8–S10: G4-FID assays. Figure S11. Obtained CD spectra for the tetracationic *opp*-dibenzoporphyrin derivatives **H_2_-β-TMePyBP, Zn-β-TMePyBP and Ni-β-TMePyBP** studied.

## Sample Availability

Samples of the compounds **TPP**, **β-Br4TPP**, **Ni-β-Br4**, **Ni-β-TPyBP**, **H_2_-β-TPyBP**, **H_2_-β-TMePyBP**, **Zn-β-TMePyBP**, and **Ni-β-TMePyBP** are available from the authors.
