# Supplementary material for: opp-Dibenzoporphyrin Pyridinium Derivatives as Potential G-Quadruplex DNA Ligands
Source: Molecules. 2023 Aug 29;28(17):6318. doi: 10.3390/molecules28176318 (PMC10489911; doi:10.3390/molecules28176318)
Supplement: Supplementary file 1 [file molecules-28-06318-s001.zip › molecules-2529229-supplementary.pdf]

Supporting information

opp-Dibenzoporphyrin pyridinium derivatives as potential G-quadruplex DNA ligands

Nuno M. M. Moura*, José A. S. Cavaleiro, Maria Graça P. M. S. Neves, Catarina I. V. Ramos*

LAQV-REQUIMTE, Department of Chemistry, University of Aveiro, 3810-193 Aveiro, Portugal

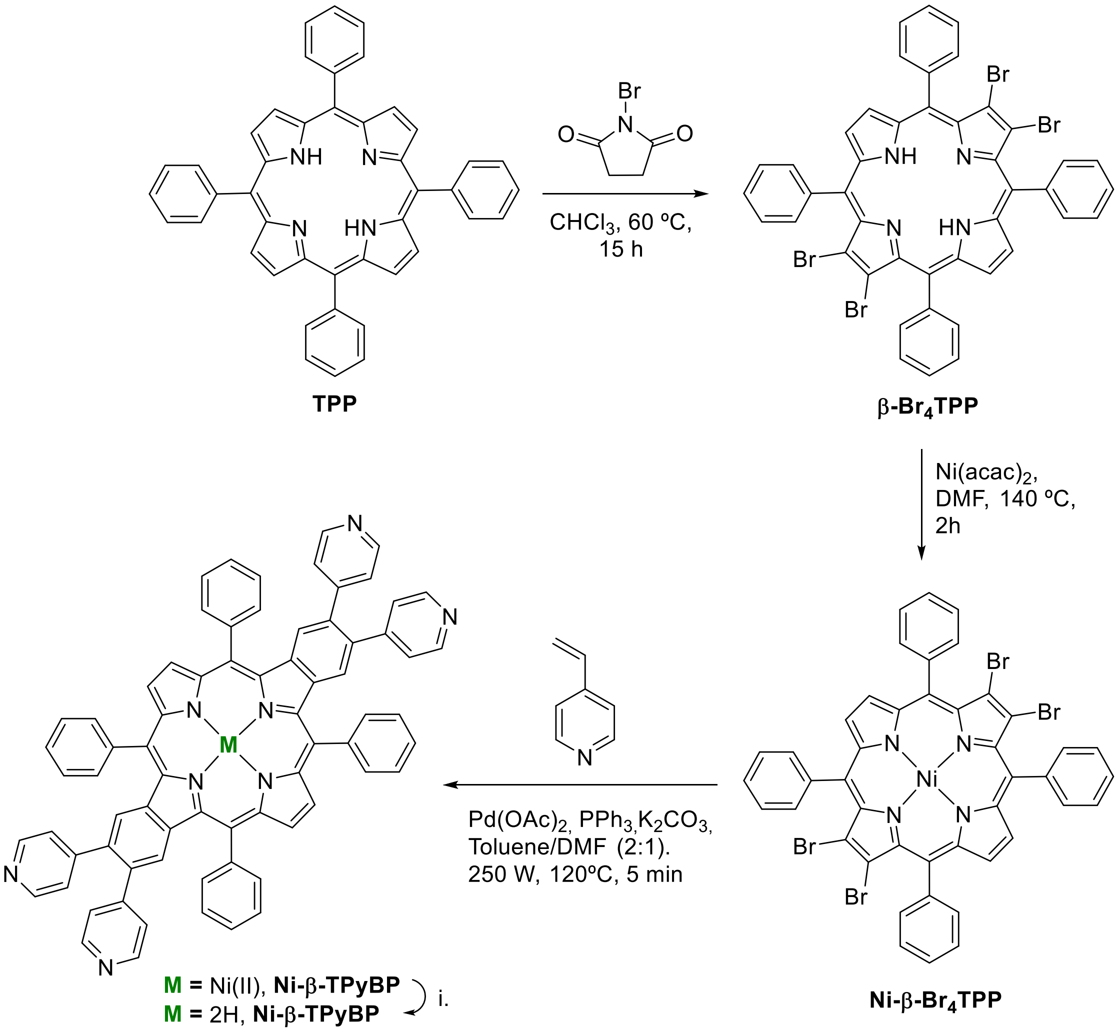

i. 10% H_2SO_4, $CHCl_3$, r.t., 20 min.

Scheme S1. Synthetic route to prepare the neutral Ni(II) complex of *opp*-dibenzoporphyrin **Ni-β-TPyBP**.

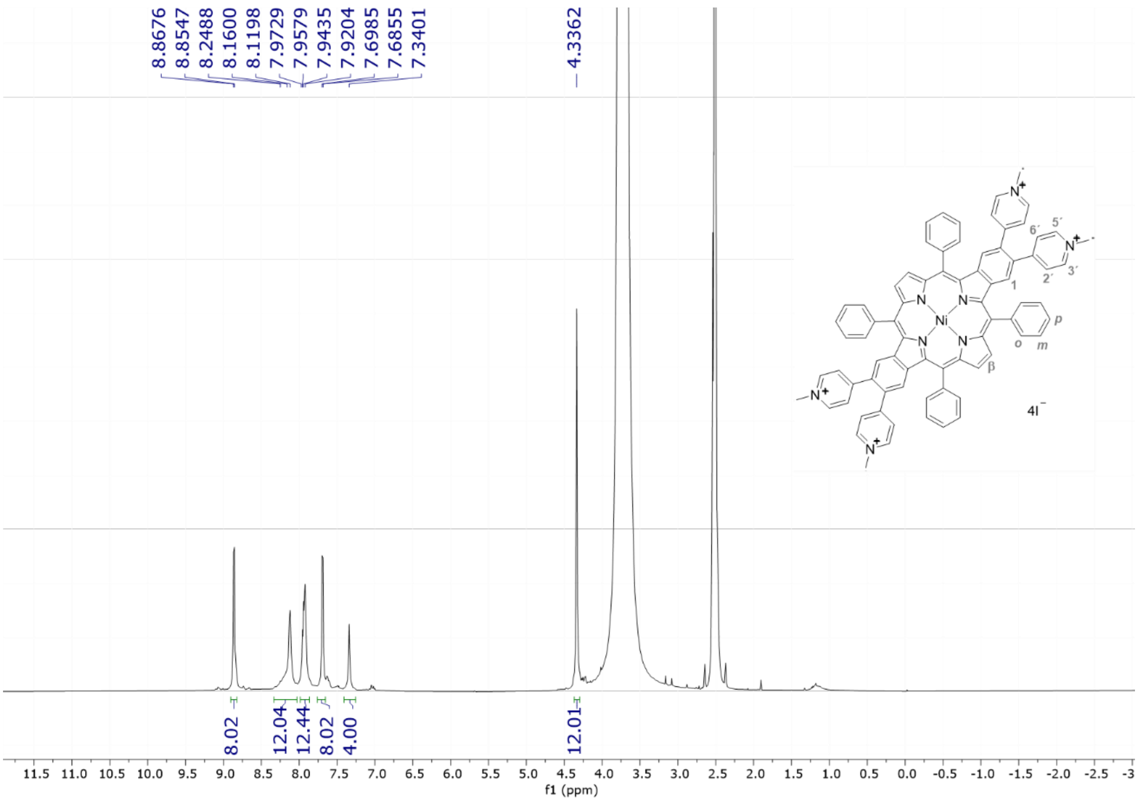

Figure S1. ^{1}H NMR spectrum of **Ni-β-TMePyBP** in DMSO-d_6.

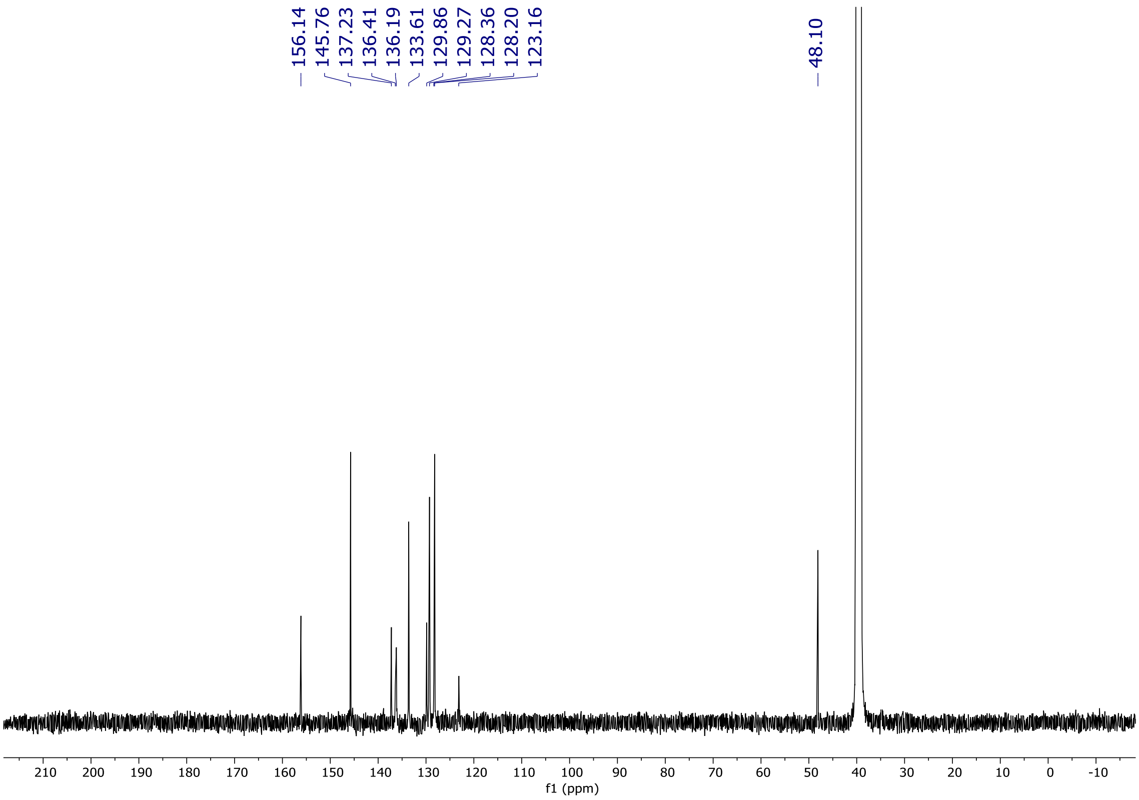

Figure S2. ^{13}C NMR spectrum of **Ni-β-TMePyBP** in DMSO-d_6.

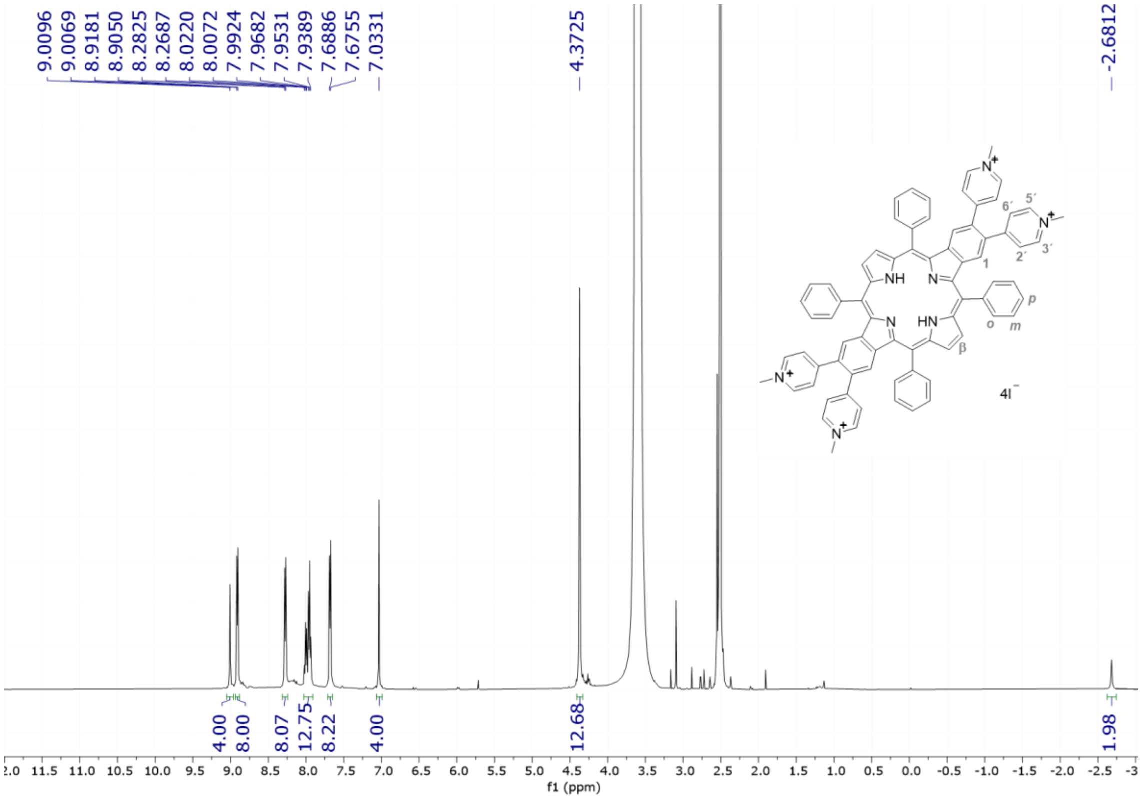

Figure S3. ^{1}H NMR spectrum of **2H-β-TMePyBP** in DMSO-d_6.

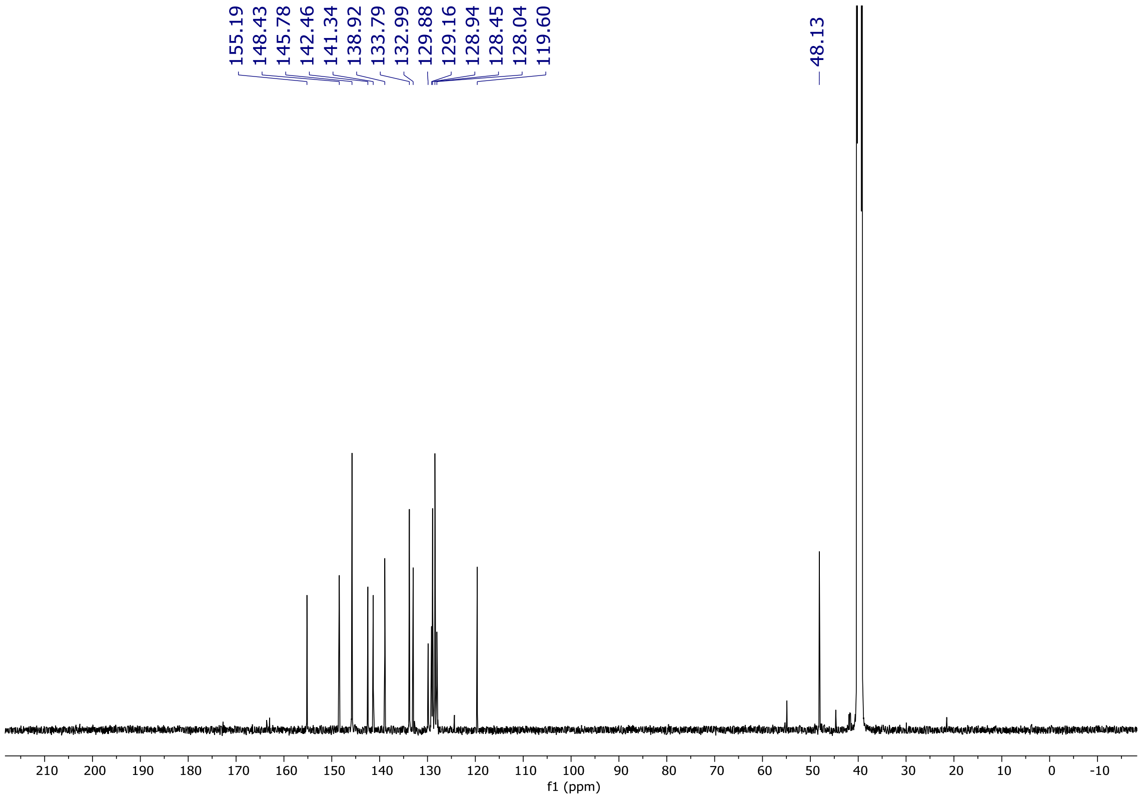

Figure S4. ^{13}C NMR spectrum of **2H-β-TMePyBP** in DMSO-d_6.

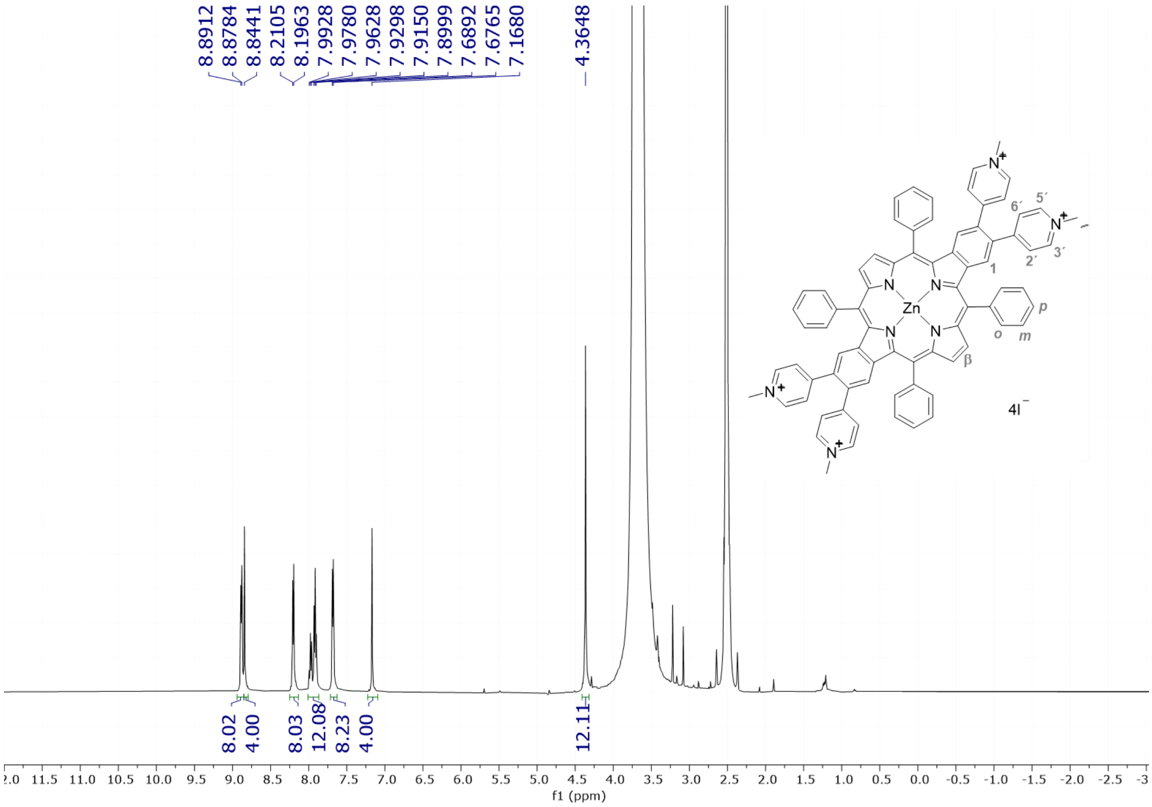

Figure S5. ^{1}H NMR spectrum of **Zn-β-TMePyBP** in DMSO-d_6.

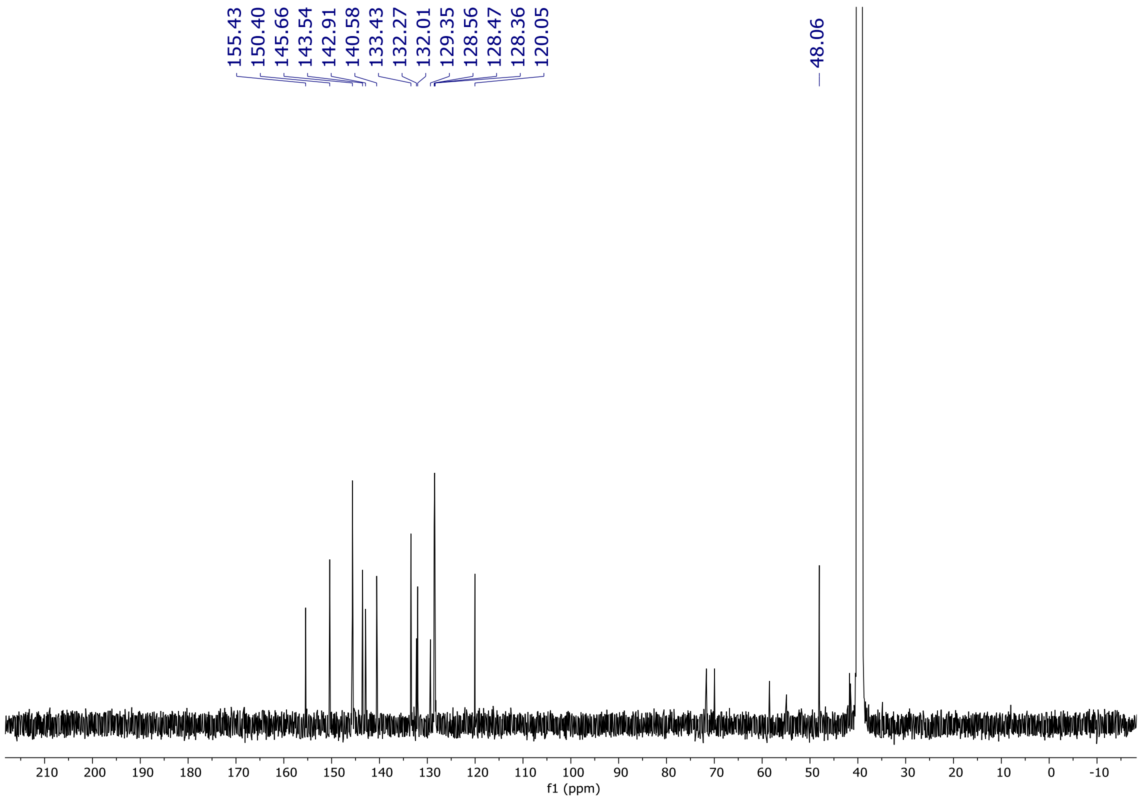

Figure S6. ^{13}C NMR spectrum of **Zn-β-TMePyBP** in DMSO-d_6.

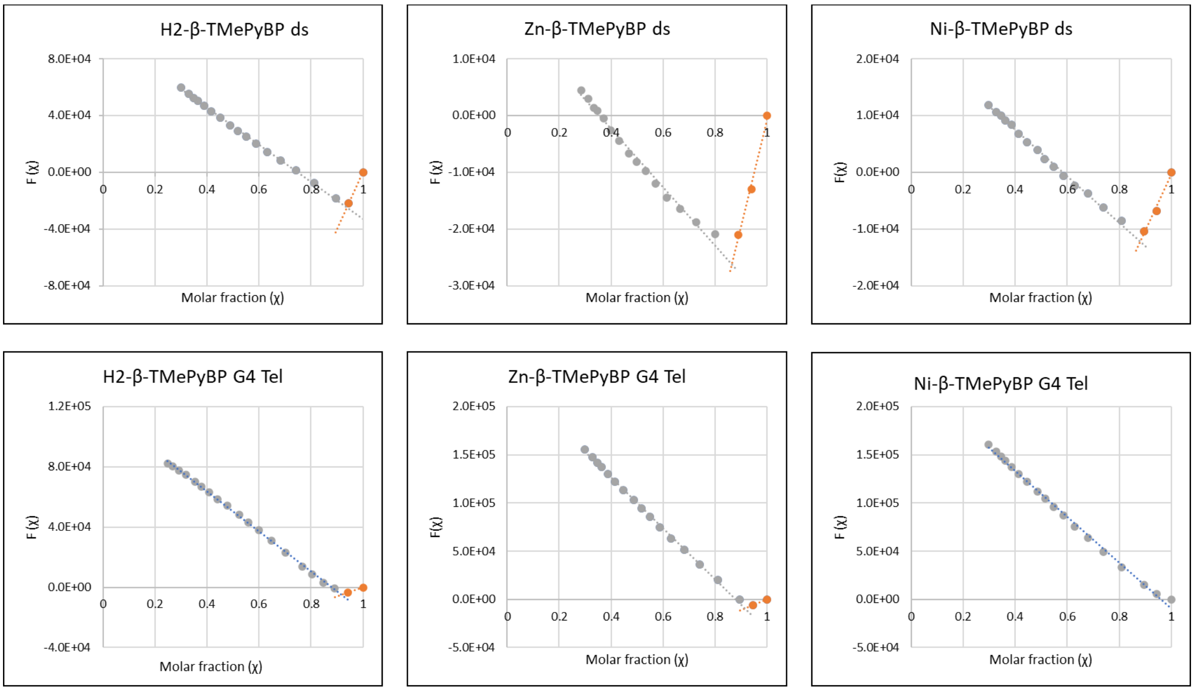

Figure S7. Job plot obtained from the different UV-Vis titrations.

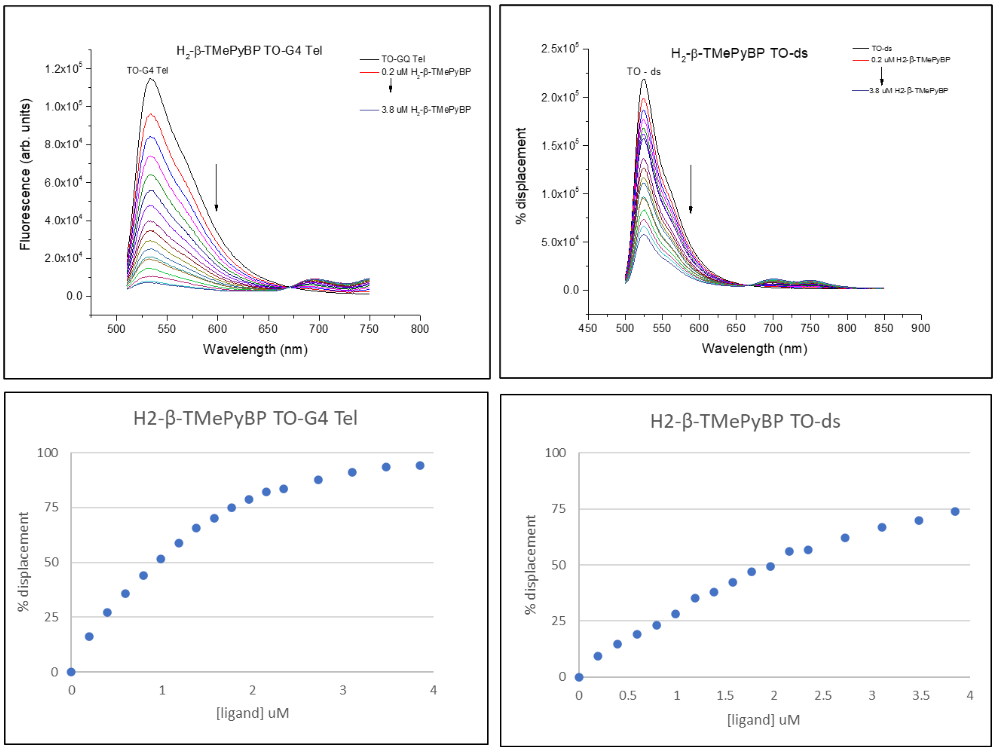

Figure S8. Results from G4-FID obtained for the ligand **H2-β-TMePyBP**

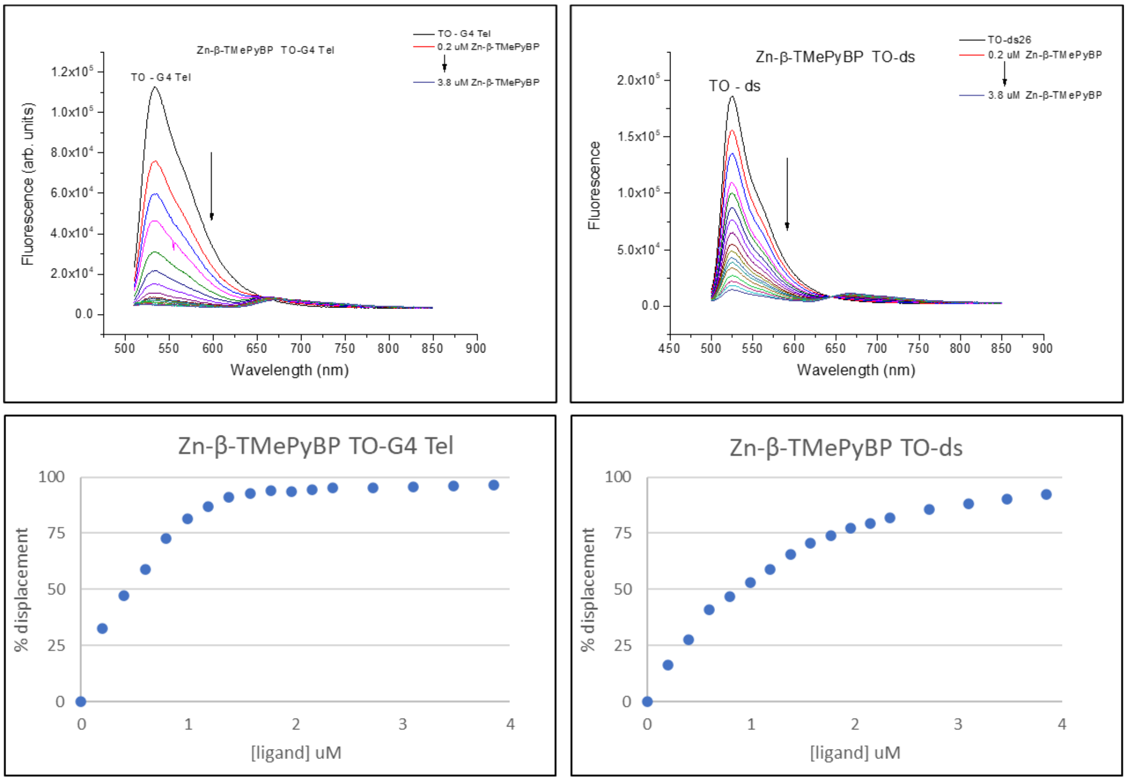

Figure S9. Results from G4-FID obtained for the ligand **Zn-β-TMePyBP**

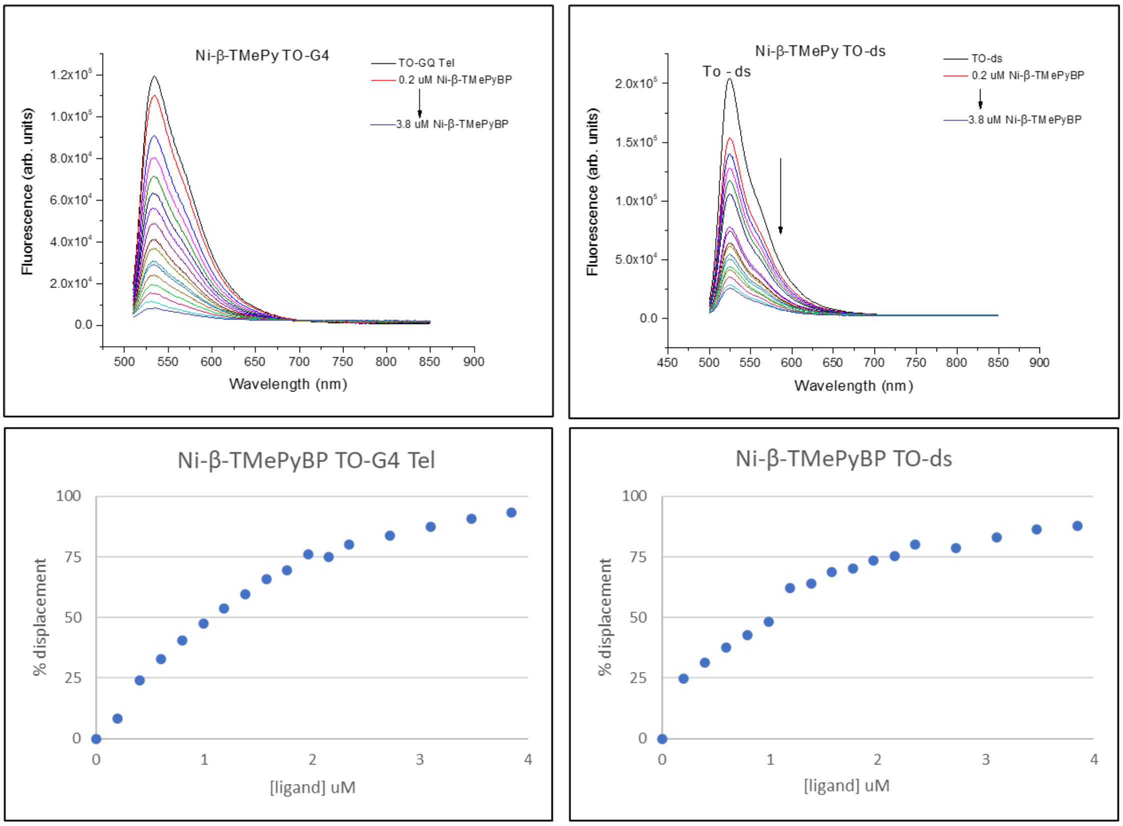

Figure S10. Results from G4-FID obtained for the ligand **Ni-β-TMePyBP**

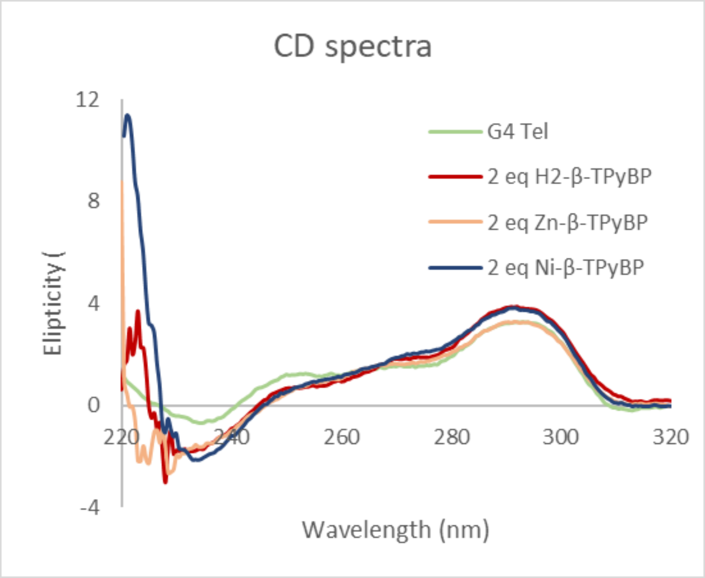

Figure S11. Obtained CD spectra for the tetracationic *opp*-dibenzoporphyrin derivatives **H2-β-TMePyBP**, **Zn-β-TMePyBP** and **Ni-β-TMePyBP** studied.
